# Meta‐analysis of the influence of a positive circumferential resection margin in oesophageal cancer

**DOI:** 10.1002/bjs5.50183

**Published:** 2019-06-25

**Authors:** R. Evans, J. R. Bundred, P. Kaur, J. Hodson, E. A. Griffiths

**Affiliations:** ^1^ Department of Upper Gastrointestinal Surgery University Hospitals Birmingham NHS Foundation Trust Birmingham UK; ^2^ Institute of Translational Medicine University Hospitals Birmingham NHS Foundation Trust Birmingham UK; ^3^ College of Medical and Dental Sciences University of Birmingham Birmingham UK; ^4^ Institute of Cancer and Genomic Sciences, College of Medical and Dental Sciences University of Birmingham Birmingham UK

## Abstract

**Background:**

The evidence regarding the prognostic impact of a positive circumferential resection margin (CRM) in oesophageal cancer is conflicting, and there is global variability in the definition of a positive CRM. The aim of this study was to determine the impact of a positive CRM on survival in patients undergoing oesophagectomy for oesophageal cancer.

**Methods:**

A systematic review and meta‐analysis was performed. PubMed and Embase databases were searched for articles to May 2018 examining the effect of a positive CRM on survival. Cohort studies written in English were included. Meta‐analyses of univariable and multivariable hazard ratios (HRs) were performed using both Royal College of Pathologists (RCP) and College of American Pathologists (CAP) criteria. Risk of bias was assessed using the Newcastle–Ottawa Scale. Egger regression, and Duval and Tweedie trim‐and‐fill statistics were used to assess publication bias.

**Results:**

Of 133 studies screened, 29 incorporating 6142 patients were finally included for analysis. Pooled univariable HRs for overall survival in patients with a positive CRM were 1·68 (95 per cent c.i. 1·48 to 1·91; *P* < 0·001) and 2·18 (1·84 to 2·60; *P* < 0·001) using RCP and CAP criteria respectively. Subgroup analyses demonstrated similar results for patients by T category, neoadjuvant therapy and tumour type. Pooled HRs from multivariable analyses suggested that a positive CRM was independently predictive of a worse overall survival (RCP: 1·41, 1·21 to 1·64, *P* < 0·001; CAP: 2·37, 1·60 to 3·51, *P* < 0·001).

**Conclusion:**

A positive CRM is associated with a worse prognosis regardless of classification system, T category, tumour type or neoadjuvant therapy.

## Introduction

The incidence of oesophageal adenocarcinoma has increased steadily over the past 30 years and now accounts for up to one‐sixth of cancer‐related mortality^1^. Owing to its aggressive nature, late symptomatology, early haematological and lymphatic spread, curative surgery remains a treatment for selected patients only, with significant risks of morbidity and mortality^2^, ^3^. It is therefore important to select good candidates for surgery. Lymph node involvement, depth of tumour invasion, and distal and proximal resection margin involvement have been shown consistently to be associated with a poor prognosis in patients who undergo resection^4^, ^5^, ^6^, ^7^, ^8^, ^9^, ^10^.

Most authors^11^, ^12^ have reported that circumferential resection margin (CRM) involvement is associated with an increased risk of local recurrence and poorer long‐term survival in oesophageal cancer. This association, however, was not observed in all studies^13^, ^14^, ^15^. This discrepancy may be explained partly by the ongoing dispute regarding the exact pathological classification of CRM involvement. The Royal College of Pathologists (RCP) in the UK defines positive CRM as cases where tumour is found within 1 mm of the surgical margin, whereas the College of American Pathologists (CAP) defines CRM as positive if tumour is found at the cut margin of resection (a 0‐mm margin). The optimal definition of a positive CRM remains unknown. In addition, many studies have had a relatively small sample size and short follow‐up^12^, ^16^. These factors may have biased the true effect of a positive CRM on outcomes.

A former meta‐analysis^17^ on this topic from 2014 comprised 19 studies published between 1993 and 2013, and concluded that a positive CRM according to either the RCP or the CAP definition was associated with a poor prognosis. Since then, there have been advancements in neoadjuvant and adjuvant treatment regimens, and further studies^8^, ^13^, ^14^, ^15^, ^18^, ^19^, ^20^, ^21^, ^22^, ^23^, ^24^, ^25^, ^26^, ^27^, ^28^, ^29^, ^30^, ^31^, ^32^, ^33^, ^34^, ^35^, ^36^, ^37^, ^38^, ^39^, ^40^, ^41^, ^42^, ^43^, ^44^ examining CRM status in oesophageal cancer have been published. The aim of the present systematic review and meta‐analysis was to examine further the role of CRM status in the current era of multimodality treatment of patients with oesophageal cancer.

## Methods

Patients undergoing oesophagectomy for oesophageal cancer formed the population for this meta‐analysis. CRM‐positive and ‐negative pathology specimens, defined by both CAP and RCP criteria, formed the intervention and comparator, with overall survival (OS) defined as the outcome. PRISMA guidelines^45^ were used to design the study and report the findings. The protocol of this meta‐analysis was registered in the Prospective Register of Systematic Reviews (PROSPERO identification code CRD42017078901).

### Search strategy and selection

A literature search was conducted using MEDLINE (PubMed) and Embase online databases, using the search terms ‘oesophageal/esophageal cancer’ or ‘oesophageal/esophageal carcinoma’ or ‘oesophageal/esophageal neoplasm’ or ‘oesophagectomy/esophagectomy’, and ‘circumferential resection margin’ or ‘radial resection margin’ or ‘lateral resection margin’. The search was limited to articles in English and a publication date up to May 2018. The bibliographies of relevant studies were examined for further publications not found during initial database searching.

To be included, studies had to: report on a population of patients undergoing curative oesophagectomy for oesophageal cancer exclusively; investigate the relationship between CRM status and survival; and either report hazard ratios (HRs) for OS or present Kaplan–Meier plots from which HRs could be estimated^46^, ^47^. Reviews and case reports were excluded. Conference proceedings were also examined.

### Data extraction and outcome definitions

All eligible studies were identified by two reviewers, who independently extracted data. Data extracted included author name, year of publication, country of study, total number of patients, mean/median age, proportion of male participants, tumour T category, tumour histology, neoadjuvant treatment, definition of CRM (RCP, CAP or both), length of follow‐up (mean/median and range), as well as HRs for OS, with 95 per cent c.i. and *P* values. Any disputes were resolved by a third reviewer.

The RCP defined a positive CRM as the presence of tumour within 1 mm of the margin^12^. The CAP defined tumour at the CRM as positive^16^.

All studies were graded for methodological and reporting quality using the Newcastle–Ottawa Scale (NOS). This involves scoring the selection of patients into the study, the comparability of the two included cohorts and the assessment of outcomes. Two reviewers scored each paper and disputes were resolved by a third reviewer.

### Statistical analysis

If studies reported survival data using Kaplan–Meier curves, a HR with 95 per cent c.i. was estimated according to the method described by Parmar and colleagues^46^, with survival rates extracted from plots at yearly intervals and constant censoring assumed. Where studies stratified patients into three categories (no tumour within 1 mm of the CRM, tumour within 1 mm of the CRM but not within 0 mm of the CRM, and tumour at the CRM), these were combined by taking weighted means of the survival curves in order to estimate the HRs for RCP and CAP definitions.

The HRs of studies were then pooled using a random‐effects model with inverse variance weighting. Studies were summarized using forest plots, and *I* ^2^ statistics were calculated as measures of heterogeneity. To investigate causes of heterogeneity, the following subgroup analyses were performed: category pT3 only; adenocarcinoma or squamous cell carcinoma (SCC) only; neoadjuvant therapy; studies reporting HRs; and studies reporting data by Kaplan–Meier analysis. In addition, for studies that performed multivariable analyses, the resulting HRs for CRM were pooled to assess the independent association between CRM and survival.

Publication biases were examined using funnel plots, Begg and Mazumdar rank correlation analysis, and Egger linear regression tests. The Duval and Tweedie trim‐and‐fill method^48^ was used to impute studies to correct for publication biases.

Statistical analysis was performed using Comprehensive Meta‐Analysis® version 3 (Biostat, Englewood, New Jersey, USA) and RevMan® version 5.3 (The Cochrane Collaboration, The Nordic Cochrane Centre, Copenhagen, Denmark).

## Results

**Figure 1:**
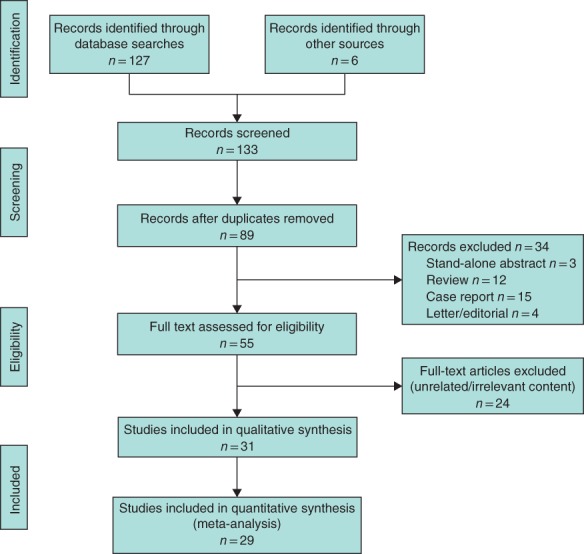
PRISMA diagram for the review

A total of 127 studies were initially found. Cross‐references led to a further six studies that were possibly eligible. After removing duplicates, initial screening and full‐text review 31 studies remained (*Fig*. 1). Some 29 studies^8^, ^13^, ^14^, ^15^, ^19^, ^21^, ^22^, ^23^, ^24^, ^25^, ^26^, ^27^, ^28^, ^29^, ^30^, ^31^, ^32^, ^33^, ^34^, ^35^, ^36^, ^37^, ^38^, ^39^, ^40^, ^41^, ^42^, ^43^, ^44^ from ten different countries, published between 2001 and 2018, were included in the meta‐analysis (*Table* S1, supporting information).

The median number of patients per study was 172 (range 59–479), with a total population of 6142 patients. Mean/median age ranged from 58 to 68 years across the studies (median 64 years), and a male preponderance was observed, with cohorts being 65–96 per cent men (median 79 per cent). There was a degree of variation across the studies of the defined histopathological inclusion criteria (*Table* S2, supporting information). Twelve studies included all histological types of oesophageal malignancy, ten included both adenocarcinoma and SCC, four included adenocarcinoma alone and three included SCC alone. Twelve studies included only patients with category T3 disease. Seventeen studies defined margins using both the RCP and CAP criteria, whereas 11 used RCP criteria alone and a single study used solely CAP criteria.

The use of neoadjuvant chemotherapy and chemoradiotherapy (CRT) varied across studies and within the studies themselves. Eight studies did not use neoadjuvant therapy, 13 administered chemotherapy only, four used CRT only, and four used both chemotherapy and CRT. Across the studies, the median rate of a positive CRM was 45·3 (range 18·2–78·3) per cent using the RCP definition and 17·6 (4·9–30·0) per cent according to the CAP definition. Reporting on the duration of patient follow‐up was sporadic and varied markedly across studies. NOS scores varied from 5 to 9 (median 7) (*Table* 1).

**Table 1:** Newcastle–Ottawa Scale grading for eligible studies

	Newcastle–Ottawa Scale grading			
Reference	Selection	Comparability	Outcome	Score
Dexter *et al*.^19^	4	0	2	6
Khan *et al*.^21^	3	1	3	7
Roh *et al*.^22^	4	1	2	7
Griffiths *et al*.^23^	3	2	2	7
Thompson *et al*.^8^	3	2	3	8
Sujendran *et al*.^24^	4	0	1	5
Deeter *et al*.^25^	3	1	2	6
Scheepers *et al*.^26^	4	0	2	6
Saha *et al*.^27^	4	1	2	7
Sillah *et al*.^28^	4	0	2	6
Mirnezami *et al*.^29^	4	1	1	6
Pultrum *et al*.^30^	3	1	2	6
Chao *et al*.^31^	3	2	2	7
Verhage *et al*.^32^	2	1	3	6
Harvin *et al*.^33^	3	2	2	7
Rao *et al*.^34^	2	0	3	5
Reid *et al*.^35^	4	1	3	8
Salih *et al*.^36^	3	1	2	6
O'Farrell *et al*.^13^	3	1	2	6
O'Neill *et al*.^37^	3	1	1	5
Ahmad *et al*.^38^	3	1	3	7
Theologou *et al*.^14^	3	2	3	8
Gilbert *et al*.^39^	4	1	2	7
Lee *et al*.^40^	3	1	1	5
Okada *et al*.^41^	2	1	3	6
Ghadban *et al*.^42^	3	1	3	7
Depypere *et al*.^43^	3	1	3	7
Quinn *et al*.^15^	4	2	3	9
Knight *et al*.^44^	3	1	3	7

### Meta‐analysis

**Figure 2:**
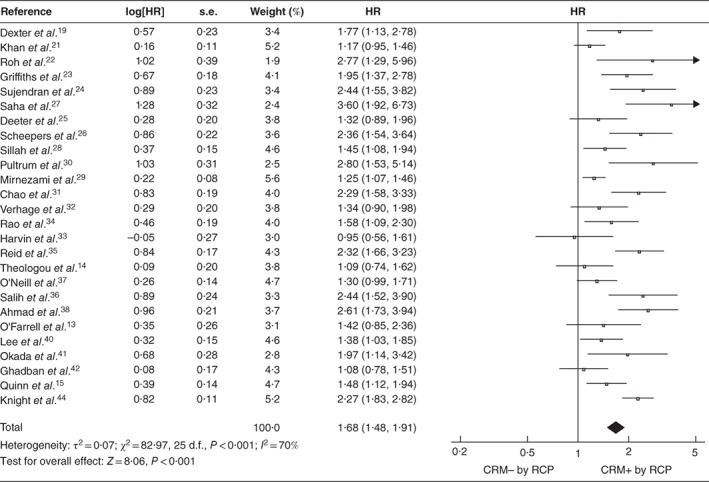
Forest plot of all studies assessing the influence of a positive circumferential resection margin in accordance with the Royal College of Pathologists' definition An inverse‐variance random‐effects model was used for meta‐analysis. Hazard ratios (HRs) are shown with 95 per cent confidence intervals. CRM−/+, negative/positive circumferential resection margin; RCP, Royal College of Pathologists.

**Figure 3:**
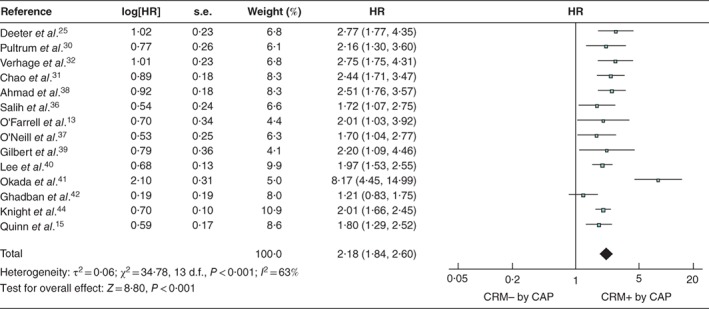
Forest plot of all studies assessing the influence of a positive circumferential resection margin in accordance with the College of American Pathologists' definition An inverse‐variance random‐effects model was used for meta‐analysis. Hazard ratios (HRs) are shown with 95 per cent confidence intervals. CRM−/+, negative/positive circumferential resection margin; CAP, College of American Pathologists.

Analysis of the 26 studies reporting univariable survival analyses, using the RCP definition, found OS to be shorter in patients with a positive CRM (pooled HR 1·68, 95 per cent c.i. 1·48 to 1·91; *P* < 0·001) (*Fig*. 2). Analysis of the 14 studies that used the CAP definition returned a similar result: pooled HR 2·18 (1·84 to 2·60; *P* < 0·001) (*Fig*. 3). Significant heterogeneity between studies was observed, with *I* ^2^ values of 70 and 63 per cent for the RCP and CAP definitions respectively.

### Publication bias

**Figure 4:**
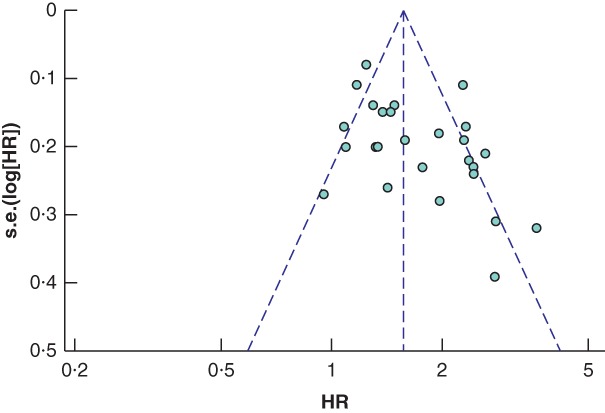
Funnel plot of all studies assessing the influence of a positive circumferential resection margin in accordance with the Royal College of Pathologists' definition HR, hazard ratio.

**Figure 5:**
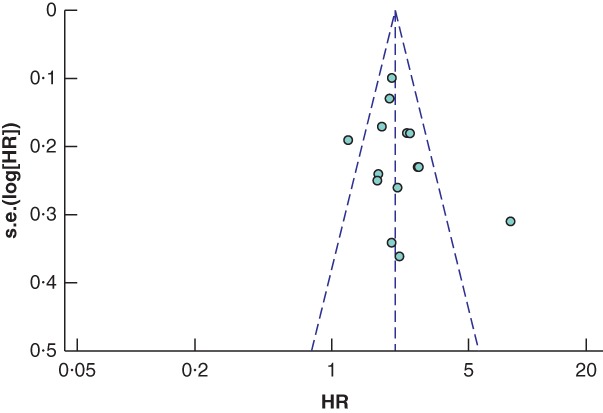
Funnel plot of all studies assessing the influence of a positive circumferential resection margin in accordance with the College of American Pathologists' definition HR, hazard ratio.

Potential publication bias was detected in the studies using the RCP definition of a positive CRM (*Fig*. 4), with significance on the Begg and Mazudmar rank test (*P* = 0·013) and Egger's test of the intercept (*P* < 0·001). In an attempt to correct for this, the Duval and Tweedie trim‐and‐fill approach was used, which identified a potential eight missing studies with effect sizes below that of the pooled HR. After accounting for these, the difference in survival between the CRM‐positive and ‐negative groups remained significant, with an imputed point estimate HR of 1·37 (95 per cent c.i. 1·19 to 1·58). Assessment of the studies reporting outcomes using the CAP definition of a positive CRM found minimal publication bias (*Fig*. 5), with a non‐significant Begg and Mazudmar rank test (*P* = 0·213) and Egger's test of the intercept (*P* = 0·132).

### Subgroup analyses

Subgroup analysis of studies reporting OS for category T3 tumours led to a pooled HR of 1·43 (95 per cent c.i. 1·20 to 1·71; *P* < 0·001) according to RCP and 2·25 (1·62 to 3·10; *P* < 0·001) according to CAP. Other subgroup analyses by tumour histology, use of neoadjuvant therapy and type of summary statistics reported by the study (HR or Kaplan–Meier curve) returned consistent results, all finding survival to be significantly shorter in those with a positive CRM (*Table* 2).

**Table 2:** Meta‐analysis of specific subgroups

	RCP				CAP			
	No. of studies	*I* ^2^ (%)	Pooled HR*	*P*	No. of studies	*I* ^2^ (%)	Pooled HR*	*P*
**Univariable analysis**								
All studies	26	70	1·68 (1·48, 1·91)	< 0·001	14	63	2·18 (1·84, 2·60)	< 0·001
pT category								
pT3 only	10	49	1·43 (1·20, 1·71)	< 0·001	8	81	2·25 (1·62, 3·10)	< 0·001
Histological type								
AC	4	81	1·78 (1·11, 2·86)	0·001	2	35	2·19 (1·67, 2·88)	< 0·001
SCC	3	13	1·79 (1·27, 2·54)	0·001	3	89	3·20 (1·67, 6·15)	< 0·001
Neoadjuvant therapy								
Yes	7	50	2·03 (1·56, 2·64)	< 0·001	4	71	2·99 (1·68, 5·33)	< 0·001
No	9	45	1·48 (1·25, 1·75)	< 0·001	3	51	2·85 (1·91, 4·24)	< 0·001
HR estimation								
Derived from Kaplan–Meier curves	14	66	1·74 (1·58, 1·91)	< 0·001	6	0	2·12 (1·84, 2·45)	< 0·001
Reported	12	69	1·52 (1·27, 1·82)	< 0·001	8	78	2·25 (1·66, 3·06)	< 0·001
**Multivariable analysis** †								
All studies	13	41	1·41 (1·21, 1·64)	< 0·001	8	80	2·37 (1·60, 3·51)	< 0·001

Values in parentheses are 95 per cent confidence intervals.

* Pooled hazard ratios (HRs) are derived from random‐effects meta‐analysis models and are for patients with a positive circumferential resection margin (CRM) relative to those with a negative CRM.

† Meta‐analysis of HRs reported from multivariable analyses; the factors accounted for in the multivariable models for the included studies are summarized in *Table* 3. RCP, Royal College of Pathologists; CAP, College of American Pathologists; AC, adenocarcinoma; SCC, squamous cell carcinoma.

### Multivariable analyses

**Figure 6:**
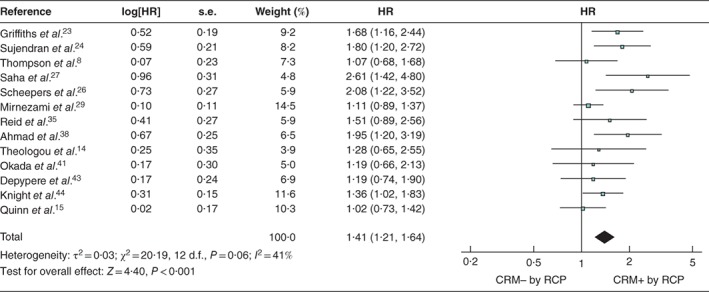
Forest plot of all studies assessing, via multivariable analysis, the influence of a positive circumferential resection margin in accordance with the Royal College of Pathologists' definition An inverse‐variance random‐effects model was used for meta‐analysis. Hazard ratios (HRs) are shown with 95 per cent confidence intervals. CRM−/+, negative/positive circumferential resection margin; RCP, Royal College of Pathologists.

**Figure 7:**
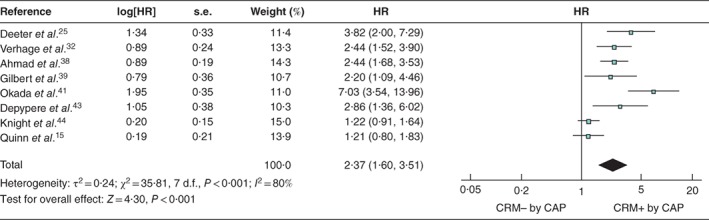
Forest plot of all studies assessing, via multivariable analysis, the influence of a positive circumferential resection margin in accordance with the College of American Pathologists' definition An inverse‐variance random‐effects model was used for meta‐analysis. Hazard ratios (HRs) are shown with 95 per cent confidence intervals. CRM−/+, negative/positive circumferential resection margin; CAP, College of American Pathologists.

The variables most commonly adjusted for in the multivariable models of included studies were node status, presence of lymphovascular invasion, tumour grade, and patient age and sex (*Table* 3). After accounting for these factors, a positive CRM remained significantly associated with shorter survival across the 13 studies using the RCP definition (pooled HR 1·41, 95 per cent c.i. 1·21 to 1·64; *P* < 0·001) (*Fig*. 6) and the eight studies that used the CAP definition (pooled HR 2·37, 1·60 to 3·51; *P* < 0·001) (*Fig*. 7).

**Table 3:** All factors included in multivariable analyses alongside circumferential resection margin status

	No. of studies including factor in multivariable analysis	
	RCP (*n* = 13)	CAP (*n* = 8)
**Demographic factors**		
Increasing age	7	3
Male sex	5	2
Worsening preoperative lung function	1	0
**Staging**		
Increasing T category	5	1
Increasing N category	12	6
LVI+	7	6
**Operative factors**		
Operation type (transhiatal *versus* transthoracic)	1	1
**Pathology**		
Positive CRM	13	8
Tumour subtype (adenocarcinoma)	3	0
Increasing tumour grade	4	3
Increasing Mandard score	1	1
Increasing tumour diameter	0	1
Increasing tumour length	0	1
Presence of Barrett's mucosa	0	1
**Concurrent treatment**		
No neoadjuvant therapy	4	3
No adjuvant therapy	2	1
Preoperative stenting	1	1

RCP, Royal College of Pathologists; CAP, College of American Pathologists; LVI+, presence of lymphovascular invasion; CRM, circumferential resection margin.

## Discussion

This study showed that a positive CRM, as defined by both RCP and CAP definitions, was associated with a worse overall prognosis. This association was also observed in the subgroups of patients with category T3 disease, adenocarcinoma and SCC, and in those receiving neoadjuvant therapy. Both RCP and CAP criteria for a positive CRM were associated with decreased survival, but patients positive according to CAP criteria appeared to have worse OS. Previous studies have suggested that this may be due to the effect of a positive CRM on distant recurrence. Chao and colleagues^31^ found that, although both RCP and CAP criteria defined a subgroup at increased risk of local recurrence, CAP criteria also defined a subgroup that was likely also to have distant recurrence, which markedly shortens OS in this group. Another theory is that close and involved CRM margins are a proxy for more aggressive tumour phenotypes. This effect was seen in a recent study^44^ that stratified patients by distance of tumour from the CRM, finding that as the distance to the CRM increased so did patient survival.

In the meta‐analysis of HRs of multivariable analyses, both RCP and CAP criteria still defined subgroups with worse prognosis, suggesting that these definitions are predictive of poorer survival independently of other factors, such as lymph node metastasis. This result seems logical, but must be considered with caution as the methodology and inclusion criteria of the individual studies were variable.

This review contained thorough synthesis of up‐to‐date literature, building on work by Wu and co‐workers^17^. Nine papers on the influence of a positive CRM on survival were added, including one that was not included by Wu *et al*.^17^, despite prior publication, and eight more recent publications^14^, ^15^, ^22^, ^39^, ^40^, ^41^, ^42^, ^43^, ^44^. The present study had several limitations. It is increasingly recognized that adenocarcinoma and SCC of the oesophagus are completely different disease entities with differing risk factors, treatment options, genetic basis and prognosis, and should therefore be analysed separately^49^. Although reporting in the additional papers improved over time, mixed analyses of different histological types of malignancy, without reporting of the effect of either subgroup on prognosis, is still commonplace^14^, ^38^, ^39^, ^42^. A subgroup analysis was conducted in the present study, but this was limited by the lack of separate reporting by histological type in each paper. Achieving a clear CRM in proximal and middle third tumours, which are usually SCC, is difficult, as the margins consist of the airways and major mediastinal vessels. In the lower third of the oesophagus, the CRM consists of the diaphragmatic crura and pericardial fat, which can be resected more easily. In subgroup analysis, a positive CRM was prognostic in both SCC and adenocarcinoma. In future studies in this area, SCC and adenocarcinoma should be analysed as separate groups to avoid introducing confounding variables.

A further limitation is that oncological and surgical treatment modalities for oesophageal cancer have changed over time. The increase in patients receiving chemotherapy and CRT over the review period has the potential to reduce the positive CRM rate, in particular with the recent introduction of fluorouracil/leucovorin, oxaliplatin and docetaxel (FLOT)‐style neoadjvuant chemotherapy, which has a high complete pathological response rate^50^, ^51^. Likewise, operative techniques have developed, with an increasing number of minimally invasive oesophagectomies performed. Studies^15^, ^44^ assessing CRM status and minimally invasive surgical techniques have not, however, shown any differences in oncological adequacy, so far.

Patients with T1 or T2 disease do not have a positive CRM, unless the resectional field has been violated. Therefore, papers with high numbers of patients with T1 or T2 tumours may have falsely inflated the prognostic role of CRM status. For this reason, a predefined subgroup analysis only of patients with category T3 tumours was undertaken. A positive CRM was still found to have a significant negative prognostic impact on OS. Several recent papers^13^, ^14^, ^37^, ^38^, ^40^, ^41^, ^42^ have examined exclusively the effect of CRM positivity on prognosis in patients with T3 disease.

Positive margins after surgery in patients undergoing neoadjuvant therapy and in those with multiple lymph node‐positive disease are known to carry a poor prognosis. The impact of neoadjuvant therapy and lymph node status as confounders of the predictive value of a positive CRM is, however, uncertain. Lymph node status is critical to survival in oesophageal cancer, and some studies have tried to account for this in reporting the influence of CRM status. One relatively small study^42^ found that CRM status was predictive of OS and locoregional recurrence, but not independently of lymph node status. In addition, another study^23^ found that a positive CRM was associated with decreased survival only in patients with a lower lymph node burden (less than 25 per cent), suggesting that a positive CRM has greater prognostic influence in those with lower stage disease. Unfortunately, owing to limited reporting in other studies^13^, ^14^, ^15^, ^29^, ^33^, ^42^, it was not possible to perform subgroup analyses of patients stratified by lymph node status, overall stage or other possible confounders, and so future research into the relationship between these variables and the CRM is warranted.

## Supporting information


**Table S1** Key characteristics and demographics of eligible studies
**Table S2** Pathology and outcome data for eligible studies
