# Supplementary material for: Meta‐analysis of the influence of a positive circumferential resection margin in oesophageal cancer
Source: BJS Open. 2019 Jun 25;3(5):595–605. doi: 10.1002/bjs5.50183 (PMC6773635; doi:10.1002/bjs5.50183)
Supplement: Supplementary file 1 — Table S1 Key characteristics and demographics of eligible studies Table S2 Pathology and outcome data for eligible studies [file BJS5-3-595-s001.docx]

**BJS5_50183**

**Meta-analysis of the influence of a positive circumferential resection margin in oesophageal cancer**

**R. Evans, J. R. Bundred, P. Kaur, J. Hodson and E. A. Griffiths**

**Table S1 Key characteristics and demographics of eligible studies**

| **Study** | **No. of** | **Average age** | **Male (%)** | **Histology** | **Neoadjuvant** |
| --- | --- | --- | --- | --- | --- |
|  | **patients** | **(years)** |  |  | **Therapy (*N)*** |
| **Dexter et al^(19)^** | 135 | 64 | 65 | AC/SCC | None |
| **Khan et al ^(21)^** | 329 | 65 | 66 | AC/SCC | None |
| **Roh et al ^(22)^** | 59 | 60 | 90 | AC/SCC/Other | None |
| **Griffiths et al ^(23)^** | 249 | 64 | 78 | AC/SCC/Other | CT(34) |
| **Thompson et al ^(8)^** | 240 | 62 | 78 | AC/SCC | CT(124) |
| **Sujendran et al ^(24)^** | 242 | 65 | 74 | AC/SCC | CT(142),CRT(9) |
| **Deeter et al ^(25)^** | 135 | 60 | 85 | AC/SCC/Other | CT(59) |
| **Scheepers et al ^(26)^** | 110 | 64 | 73 | AC/SCC | CT(31) |
| **Saha et al ^(27)^** | 105 | 61 | 83 | AC | CT(105) |
| **Sillah et al ^(28)^** | 320 | - | - | AC/SCC/Other | None |
| **Mirnezami et al ^(29)^** | 314 | 63 | 79 | AC/SCC | None |
| **Pultrum et al ^(30)^** | 98 | 65 | 78 | AC/SCC/Other | None |
| **Chao et al ^(31)^** | 151 | 58 | 96 | SCC | CRT(151) |
| **Verhage et al ^(32)^** | 132 | 63 | 85 | AC | None |
| **Harvin et al ^(33)^** | 160 | 60 | 92 | AC | CRT(160) |
| **Rao et al ^(34)^** | 115 | 64 | 66 | AC/SCC | CT (37) |
| **Reid et al ^(35)^** | 269 | - | 77 | AC/SCC | CT(24), CRT(42) |
| **Salih et al ^(36)^** | 232 | 62 | 76 | AC/SCC/Other | CT(232) |
| **O’Farrell et al ^(13)^** | 157 | 63 | 69 | AC/SCC/Other | CRT(82) |
| **O’Neill et al ^(37)^** | 226 | 64 | 81 | AC/SCC | CT (130) |
| **Ahmad et al ^(38)^** | 195 | 66 | 71 | AC/SCC/Other | CT (75) |
| **Theologou et al ^(14)^** | 199 | 66.5 | 68.4 | AC/SCC/Other | CT(118) |
| **Gilbert et al ^(39)^** | 154 | 63^+ve^, 66^-ve^ | 90^+ve^,74^-ve^ | AC/SCC/Other | CT (22), CRT (35) |
| **Lee et al ^(40)^** | 479 | 64 | 94.6 | SCC | CT/CRT (78) |
| **Okada et al ^(41)^** | 160 | 68 | 80.6 | SCC | CT (67) |
| **Ghadban et al ^(42)^** | 180 | 61 | 79.4 | AC/SCC | None |
| **Depypere et al ^(43)^** | 163 | 61 | 80 | AC/SCC | CRT(163) |
| **Quinn et al ^(15)^** | 390 | 67.3 | 79 | AC/SCC/Other | CT (298) |
| **Knight et al ^(44)^** | 444 | 62 | 85 | AC | CT(338) |

*Studies are ordered by date of publication*

*Abbreviations used: +ve/-ve: positive/negative margin subgroups, AC : adenocarcinoma, SCC: squamous cell carcinoma, CT: chemotherapy, CRT: chemoradiotherapy.*

**Table S2 Pathology and outcome data for eligible studies**

| **Study** | **Pathology** | **Pathological T-stage** | **CRM** | | **Follow up (Months)** | | | **Survival** | |
| --- | --- | --- | --- | --- | --- | --- | --- | --- | --- |
|  | **guidance** |  | **Positive (%)** | |  |  |  | **Reporting*** | |
|  |  |  | ***RCP*** | ***CAP*** | **Median** | **Mean** | **Range** | **RCP** | **CAP** |
| **Dexter et al^(19)^** | RCP | T1-3 | 47.4 | - | 16 | 19 | - | SC | - |
| **Khan et al ^(21)^** | RCP | T1-3 | 20.3 | - | - | - | >60 | SC | - |
| **Roh et al ^(22)^** | RCP | T1-3 | 44.1 | - | - | 36 | 1-92 | SC | - |
| **Griffiths et al ^(23)^** | RCP | T1-3 | 31.7 | - | 70 | - | - | SC | - |
| **Thompson^(8)^** | RCP | T1-4 | 35.4 | - | 24 | - | - | HR^m^ | - |
| **Sujendran et al ^(24)^** | RCP | T1-4 | 23.1 | - | - | - | 12-92 | SC | - |
| **Deeter et al ^(25)^** | RCP/CAP | T3 | 61.5 | 11.8 | - | 37 | - | SC | SC |
| **Scheepers et al ^(26)^** | RCP/CAP | T1-3 | 38.2 | 15.5 | - | - | - | SC | - |
| **Saha et al ^(27)^** | RCP | T0-4 | 36.2 | - | 26 | - | 12-84 | HR | - |
| **Sillah et al ^(28)^** | RCP | T1-4 | 28.4 | - | - | - | - | HR | - |
| **Mirnezami et al ^(29)^** | RCP | T1-4 | 46.5 | - | - | - | 01-90 | HR | - |
| **Pultrum et al ^(30)^** | RCP/CAP | T1-4 | 47.9 | 25.5 | - | 37 | 4.7-124 | SC | SC |
| **Chao et al ^(31)^** | RCP/CAP | T3 | 51 | 17.2 | 43 | 50 | - | SC | SC |
| **Verhage et al ^(32)^** | RCP/CAP | T3 | 67.4 | 19.7 | - | 28.4 | 2.3-212.3 | HR | HR |
| **Harvin et al ^(33)^** | RCP/CAP | T3 | 26.3 | 5 | - | - | - | HR | - |
| **Rao et al ^(34)^** | RCP/CAP | T1-4 | 49.6 | 14.8 | - | - | >36 | SC | - |
| **Reid et al ^(35)^** | RCP | T1-4 | 38 | - | 88 | - | 4-157 | HR | - |
| **Salih et al ^(36)^** | RCP/CAP | T1-4 | 45.3 | 16.4 | 18 | - | 1.2-108 | SC | SC |
| **O’Farrell et al ^(13)^** | RCP/CAP | T3 | 60 | 18 | - | 22 | 03-88 | SC | SC |
| **O’Neill et al ^(37)^** | RCP/CAP | T3 | 57.5 | 20.8 | 35 | - | - | SC | HR |
| **Ahmad et al ^(38)^** | RCP/CAP | T3 | 74 | 30 | - | - | - | HR | HR |
| **Theologou et al ^(14)^** | RCP | T3 | 75.9 | - | - | 48 | 12-120 | HR | - |
| **Gilbert et al ^(39)^** | CAP | T1-4 | - | 19.5 | 14^+ve^ ,25^-ve^ | - | - | - | HR |
| **Lee et al ^(40)^** | RCP/CAP | T3 | 78.3 | 19 | - | 32 | - | HR | HR |
| **Okada et al ^(41)^** | RCP/CAP | T3 | 70.6 | 10 | - | 31 | - | HR | HR |
| **Ghadban et al ^(42)^** | RCP/CAP | T3 | 42.2 | 24.4 | - | - | - | HR | HR |
| **Depypere et al ^(43)^** | RCP/CAP | T3 | 22.7 | 4.9 | - | 42 | - | HR^m^ | HR^m^ |
| **Quinn et al ^(15)^** | RCP/CAP | T0-4 | 18.2 | 15.9 | - | - | - | HR | HR |
| **Knight et al ^(44)^** | RCP/CAP | T1-4 | 41.3 | 18 | - | - | - | SC | SC |

**The statistic used to quantify survival in the two groups: SC=survival curves, HR=hazard ratios.*

*Abbreviations Used: +ve/-ve: positive/negative margin subgroups, RCP: Royal College of Pathologists (UK), CAP: College of American Pathologists, CRM: Circumferential Resection Margin, m: multivariable hazard ratio only*
